# Complete mitochondrial genome of the hybrid of *Culter alburnus* (♀) × *Megalobrama terminalis* (♂)

**DOI:** 10.1080/23802359.2020.1772690

**Published:** 2020-06-04

**Authors:** Kai Liu, Heng-jia Ma, Xiao-yu Feng, Nan Xie

**Affiliations:** Institute of Fishery Science, Hangzhou Academy of Agricultural Sciences, Hangzhou, China

**Keywords:** Hybrid of *Culter alburnus* (♀) × *Megalobrama terminalis* (♂), mitochondrial genome, maternal inheritance

## Abstract

In this study, we determined the complete mitochondrial DNA sequence of the hybrid of *Culter alburnus* (♀) x *Megalobrama terminalis* (♂) for the first time. The complete mitochondrial genome of the hybrid was sequenced to be 16,622 bp in size following the female parent, *C. alburnus*. The genome contained 13 protein-coding genes, 22 transfer RNA genes, two ribosomal RNA genes, and two main non-coding regions (the control region and the origin of light strand replication). Sequence alignment between the mitochondrial genomes of the hybrid and its female parent showed that a total of 35 mutation sites were identified in 14 genes or regions. The genome information presented here may play an important role in further study on the genetic mechanisms of mitochondrial DNA in hybrids.

*Megalobrama terminalis* and *Culter alburnus* are two economically important fish species in the genus *Megalobrama* and *Culter*, respectively. *Megalobrama terminalis* mainly inhabited the middle and upper reaches of the Yangtze, Heilong River Basin, Qiantang River (Chen 1998). *Culter alburnus* is widely distributed in major reservoirs and lakes of China (Chen 1998). Owing to the good economic traits, *C. alburnus* has been recognized as the main aquaculture species in China (Shi et al. 2017). *Megalobrama terminalis* is more delicious flesh than *C. alburnus*, in contrast. To synthesize the economic traits of the two freshwater fish, both collected from Qiantang River (120°10′13.15″E, 30°07′11.22″N), a population of the hybrid (assigned as QZBSJF201806) of *C. alburnus* (♀) × *M. terminalis* (♂) was obtained by artificial hybridization experiment, stored at national original breeding farm of black Amur bream from Qiantang River (120°07′21.99″E, 30°08′35.53″N), and the complete mitochondrial genome in an individual of the hybrid F1 was sequenced.

The total genomic DNA of a specimen of the hybrid was extracted from the fin tissue using the phenol-chloroform extraction method (Sambrook et al. 1989). 19 pairs of PCR primers were designed to amplify the whole mitogenome sequences based on the *C. alburnus* mitochondrial nucleotide sequences (GenBank No. GU190362). PCR products were directly sequenced by Personal Gene Technology CO., Ltd (Shanghai, China) after purification. The complete nucleotide sequence after assembled had been registered in the GenBank with accession numbers of MT249227. The annotation process was undertaken using MITOFISH prediction server (Iwasaki et al. 2013).

The phylogenetic tree of the hybrid of *C. alburnus* (♀) × *M. terminalis* (♂) is shown in Figure 1, which was drawn using the software IQ-TREE (Nguyen et al. 2015). The length of the complete mitogenome sequence of the hybrid was 16,622 bp, similar to that of *C. alburnus* (GU190362). The whole mitogenome contained 22 tRNA genes, two ribosomal RNA genes, 13 protein-coding genes (PCGs), and two main non-coding regions. Most of the genes of the hybrid were encoded on heavy strand (H-strand) except for ND6 and 8 tRNA genes, which were encoded on the light strand (L-strand). Within the genome, all the 13 PCGs included the orthodox start codon ATG except for COX1. However, the stop codons of the 13 PCGs were different from TAA, TA– or T—. The origin of light strand replication (OL) which extends up to 32 nucleotides was identified in a cluster of five tRNA genes (WANCY region) between tRNA-Asn and tRNA-Cys. The second non-coding region, the control region (D-loop), was located between the tRNA-Pro and tRNA-Phe genes with 936 bp in length.

**Figure 1.**
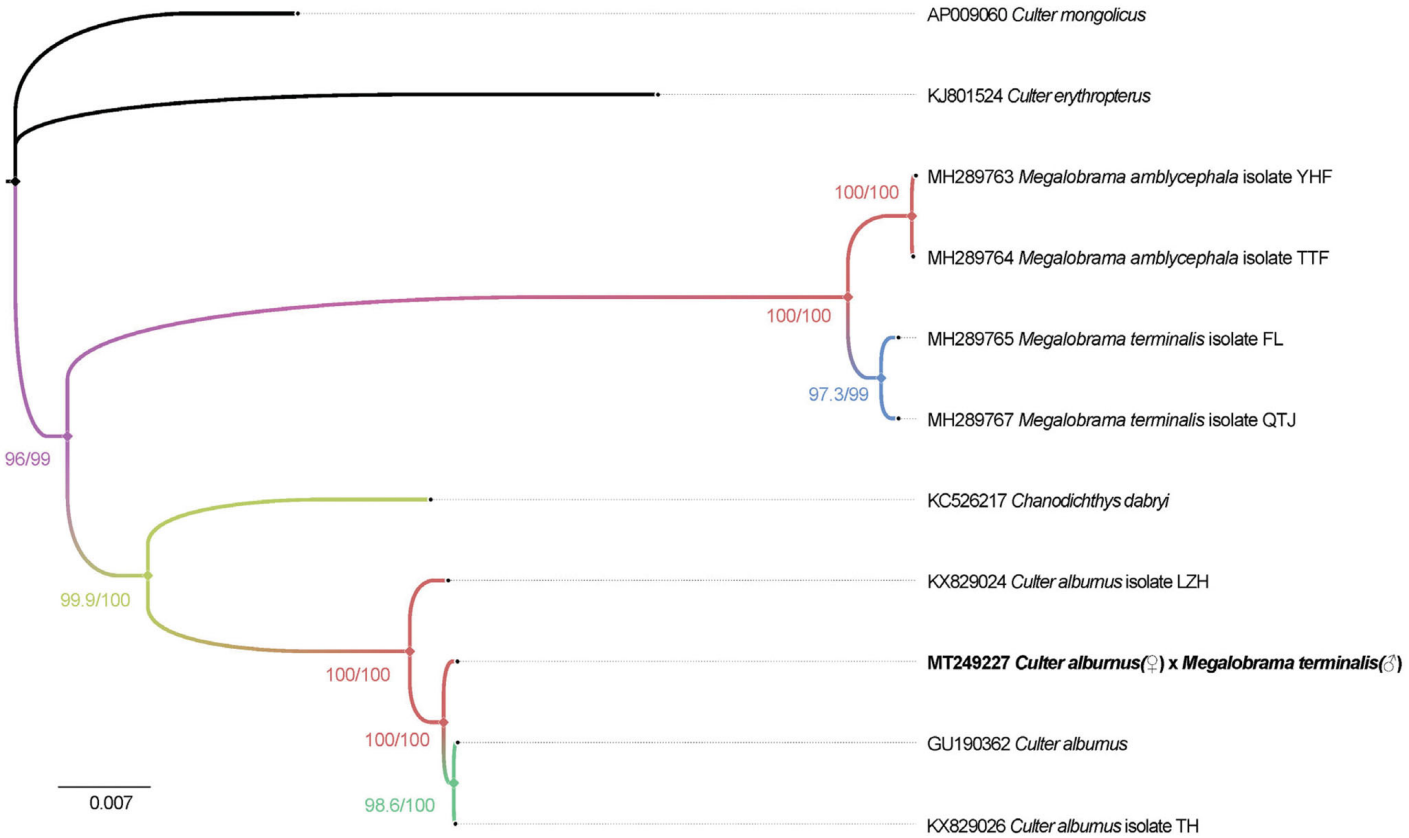
Phylogenetic tree of the hybrid of *C. alburnus* (♀) × *M. terminalis* (♂) and 10 species in the genus *Megalobrama* and *Culter* inferred by using the maximum-likelihood (ML) method based on the complete mitochondrial genome data. Values shown at each node of the tree correspond to the SH-aLRT test values and ultrafast bootstrap value given in percentages (Hoang et al. 2018).

About 99.79% sequence identity between the hybrid and *C. alburnus* (GU190362) confirmed the maternal inheritance pattern followed by the mitogenome of the hybrid. However, a total of 35 mutation sites were identified in 14 genes or regions of mitogenome of the hybrid.

## Data Availability

The data that support the findings of this study are openly available in National Center for Biotechnology Information (NCBI) at https://www.ncbi.nlm.nih.gov/nuccore/MT249227, reference number MT249227.

## References

[CIT0001] Chen YY. 1998. Fauna Sinica: Osteichthyes Cypriniformes II. Beijing: Science Press.

[CIT0002] Hoang DT, Chernomor O, von Haeseler A, Minh BQ, Vinh LS. 2018. UFBoot2: improving the ultrafast bootstrap approximation. Mol Biol Evol. 35(2):518–522. 29077904 10.1093/molbev/msx281 PMC5850222

[CIT0003] Iwasaki W, Fukunaga T, Isagozawa R, Yamada K, Maeda Y, Satoh TP, Sado T, Mabuchi K, Takeshima H, Miya M, et al. 2013. MitoFish and MitoAnnotator: a mitochondrial genome database of fish with an accurate and automatic annotation pipeline. Mol Biol Evol. 30(11):2531–2540. 23955518 10.1093/molbev/mst141 PMC3808866

[CIT0004] Nguyen L-T, Schmidt HA, von Haeseler A, Minh BQ. 2015. IQ-TREE: a fast and effective stochastic algorithm for estimating maximum-likelihood phylogenies. Mol Biol Evol. 32(1):268–274. 25371430 10.1093/molbev/msu300 PMC4271533

[CIT0005] Sambrook J, Fritsch EF, Maniatis T. 1989. Molecular cloning: a laboratory manual. 2nd ed. New York: Cold Spring Harbor Laboratory.

[CIT0006] Shi J, Wang D, Wang J, Sheng J, Peng K, Hu B, Zeng L, Xiao M, Hong Y. 2017. Comparative analysis of the complete mitochondrial genomes of three geographical topmouth culter (*Culter alburnus*) groups and implications for their phylogenetics. Biosci Biotech Bioch. 81(3):482–490. 10.1080/09168451.2016.1270739 28067596

